# Supragingival Microbial Profiles of Permanent and Deciduous Teeth in Children with Mixed Dentition

**DOI:** 10.1371/journal.pone.0146938

**Published:** 2016-01-11

**Authors:** Weihua Shi, Man Qin, Feng Chen, Bin Xia

**Affiliations:** 1 Department of Pediatric Dentistry, School of Stomatology, Peking University, Beijing, China; 2 Central Laboratory, School of Stomatology, Peking University, Beijing, China; LSU Health Sciences Center School of Dentistry, UNITED STATES

## Abstract

**Objectives:**

The present study was designed to investigate the microbial profiles of teeth in different locations in mixed-dentition-stage children, and to compare the microbiomes of permanent and deciduous teeth in the same healthy oral cavity.

**Methods:**

Supragingival plaque samples of teeth in various locations—the first permanent molars, deciduous molars, deciduous canines and incisors and permanent incisors—were collected from 20 healthy mixed-dentition-stage children with 10–12 permanent teeth erupted. Plaque DNA was extracted, and the V3–V4 hypervariable region of the bacterial 16S rRNA gene was amplified and subjected to sequencing.

**Results:**

On average, 18,051 high-quality sequences per sample were generated. Permanent tooth sites tended to host more diverse bacterial communities than those of deciduous tooth sites. A total of 12 phyla, 21 classes, 38 orders, 66 families, 74 genera were detected ultimately. Five predominant phyla (Proteobacteria, Firmicutes, Bacteroidetes, Fusobacteria and Actinobacteria) were highly variable among sites. Of 26 genera with a mean relative abundance of >0.1%, 16 showed significant differences in relative abundance among the groups. More than 20% of the total operational taxonomical units were detected only in permanent or deciduous teeth. The variation in the microbial community composition was due mainly to permanent teeth being enriched in *Actinomyces* and deciduous teeth in *Treponema*. The core microbiome of supragingival plaque in mixed dentition comprised 19 genera with complex correlationships.

**Conclusion:**

Our results suggest differences in microbial diversity and composition between permanent and deciduous teeth sites in mixed dentition. Moreover, the core microbiome of these sites was determined. These findings enhance our understanding of the development of the native oral microbiota with age.

## Introduction

The oral cavity is heavily colonized by >700 species of microorganisms [1]. These microorganisms play a vital role in oral health and disease, especially in caries and periodontal diseases [2]. It is necessary to investigate the microbial community in healthy individuals to gain a better understanding of the impact of the microbiota on oral diseases. The microbial community differs between childhood and adulthood [3, 4]. However, our understanding of the development of the native oral microbiota with age is inadequate.

To investigate the variation in the oral microbiome in children at different ages, Papaioannou *et al*. [5] used checkerboard DNA—DNA hybridization to evaluate microbial samples from three different age/dentition groups (age 3–12 years), and found that the proportions of *Actinomyces*, *Streptococcus*, *T*. *forsythia*, *P*. *gingivalis*, and *T*. *denticola* increased with age at most of the sampled locations. Crielaard *et al*. [4] also detected significant differences in the oral microbiome among children at various age stages by high-throughput sequencing. These studies suggested age-driven maturation of the oral microbiome. However, the mechanisms underlying this age-driven maturation remain unclear.

In the mixed dentition stage, deciduous teeth exfoliate successively, and new or succedaneous permanent teeth erupt. The physical changes that occur with natural growth and development can also have an influence on the oral microbiota. This is an important transitional period between childhood and adulthood for development of the microbial community. The coexistence of deciduous teeth and permanent teeth in the same oral cavity facilitates investigation of microbial development to enhance our understanding of the age-driven maturation of the oral microbiome. Most previous studies of mixed-dentition microbiota focused on pooled supragingival plaque [3] or the supragingival plaque of the first permanent teeth alone [6]. Whether the microbial profiles of deciduous and permanent teeth in the same oral cavity differ remains unclear.

The present study was designed to investigate the microbial profiles of teeth in various locations—the first permanent molars, deciduous molars, deciduous canines and incisors and permanent incisors—in mixed-dentition-stage children, and to compare the microbial communities on permanent and deciduous teeth in the same healthy oral cavity.

## Materials and Methods

### Ethics statement

The design, protocol, and informed consent of this study were approved by the Biomedical Ethics Committee of the Peking University School and Hospital of Stomatology (PKUSSIRB-201519003).

Written informed consent was obtained from the parents or guardians of all subjects in this study.

### Subject recruitment and clinical screening

Subjects were recruited from Huarun Primary School and Zhentai Primary School in Shaoshan, Hunan, China.

The clinical examination was performed in an indoor space in the schools. Due to the conditions in the two local schools, subjects were examined sitting with their back towards the examiner and then lied down on the examiner’s legs with the examiner seated behind the subject’s head. The dental examinations were performed using visual-tactile methods by the same dentist using headlamps and sterilized examination instruments. A recording clerk assisted with the recording of basic information and the findings. The dental examination was usually completed within 15 min.

**Fig 1:**
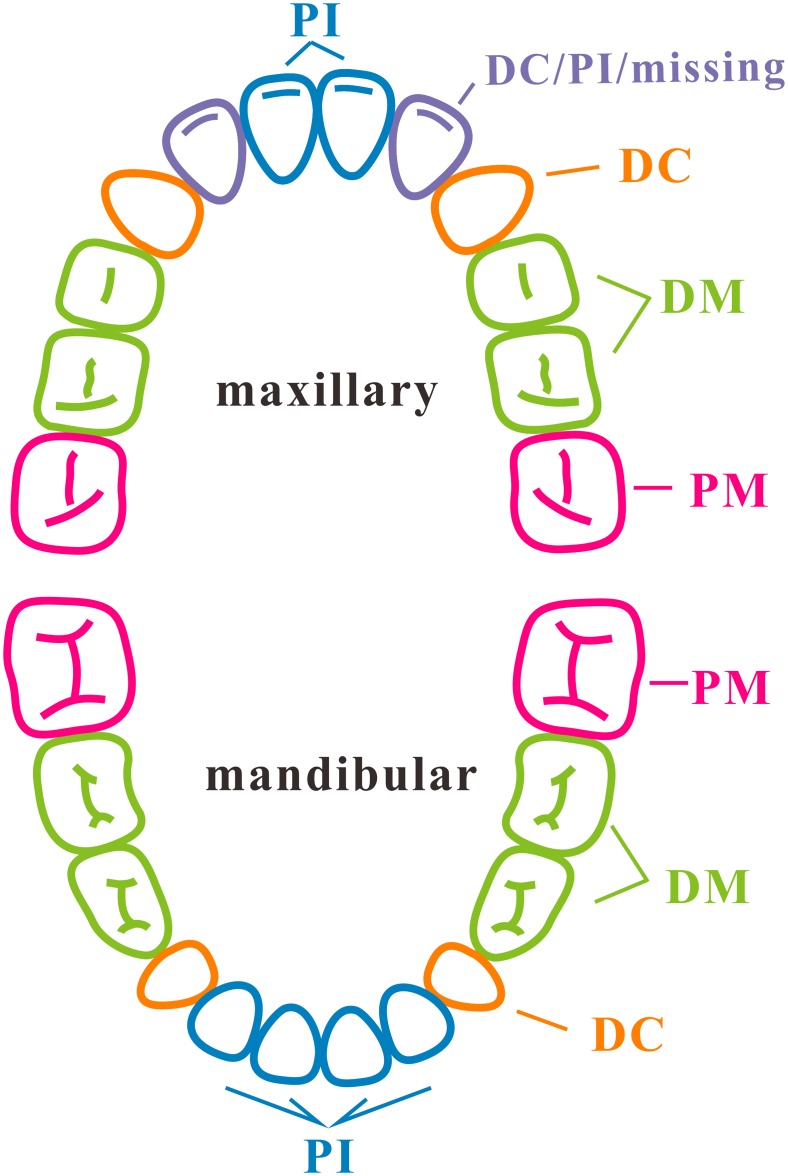
Dentition stage of the subjects, sampling tooth positions and corresponding groups. i) Dentition stage: mixed dentition stage with 10–12 fully or partially erupted permanent teeth (4 first permanent molars, 6–8 permanent incisors). ii) Sampling tooth positions and their corresponding groups: first permanent molars, PM; deciduous molars, DM; deciduous canines and incisors, DC; and permanent incisors, PI.

Inclusion criteria were that subjects (i) were medically healthy, (ii) had mixed dentition with eruption of 10–12 permanent teeth (Fig 1) and were free from dental caries (including white-spot) and dental restorations (dmf/DMF = 0), (iii) had no enamel or dentin hypoplasia detectable visually and no oral appliances, (iv) had visually healthy gingiva and had no bleeding on probing, and (v) had no antibiotic use and no fluoride treatment within the past 3 months. Caries were charted using the International Caries Detection and Assessment System (ICDAS) [7].

### Sampling

All participants were instructed to refrain from cleaning their teeth for 12 h and to avoid food and drink for 2 h before sample collection. In this study, four samples of supragingival plaque were collected separately from each subject: from the first permanent molars (PM), deciduous molars (DM), deciduous canines and incisors (DC) and permanent incisors (PI) (Fig 1). The plaque index was then recorded. Each supragingival plaque specimen was pooled from the smooth surfaces of relevant teeth in four quadrants using an individual sterile dental excavator. The plaque sample was placed in a 1.5-ml sterile centrifuge tube (Axygen, CA, USA) containing 1 ml TE buffer (10 mM Tris-HCl, 1 mM EDTA; pH 8). Specimens were collected and placed on ice immediately after sampling, transported to the local laboratory within 2 h and stored at −20°C. All samples were collected within 5 days and then conveyed on dry ice to the microbiology laboratory at Peking University School of Stomatology in Beijing within 10 h.

While sampling the supragingival plaque, the amount of dental plaque (plaque index) was assessed using the criteria of Greene and Vermillion [8].

### Microbial DNA extraction and sequencing

Microbial genomic DNA was isolated using a QIAamp DNA micro Kit (Qiagen, Hilden, Germany) in the microbiology laboratory at Peking University School of Stomatology. The procedure was performed according to the manufacturer’s instructions, with the exception of an additional lysozyme treatment (20 mg/ml, 1 h) for bacterial cell lysis [3, 9]. DNA products were evaluated by electrophoresis in 1% agarose gels run at 100 V for 30 min, and the sizes of the products (23 kb) were confirmed by comparison with a molecular size standard (S3 Fig). The final quantity and quality of the DNA were assessed by measuring the absorbance at 260 and 280 nm using an ultraviolet spectrophotometer (DU^®^ 640, Beckman Instruments, Inc., CA, USA). All DNA was stored at –20°C before further analysis.

The V3–V4 hypervariable regions of the 16S rRNA gene were subjected to high-throughput sequencing by Beijing Auwigene Tech, Ltd (Beijing, China) using the Illumina Miseq PE300 sequencing platform (Illumina, Inc., CA, USA).

PCR amplification of the V3-V4 region of Bacterial 16S rRNA gene was performed using universal primers 338F (5’-GTACTCCTACGGGAGGCAGCA-3’) and 806R (5’-GTGGACTACHVGGGTWTCTAAT-3’) [10] incorporating a sample barcode sequences. The PCR condition is as follows: 5 min initial denaturation at 95°C; 25 cycles of denaturation at 95°C (30s), annealing at 56°C (30s), elongation at 72°C (40s); and final extension at 72°C for 10min. The PCR products were separated by 1% agarose gel electrophoresis and the about 460bp fragment were purified by using Agencourt AMPure XP (Beckman Coulter, Inc., CA, USA). Sequencing were performed using Illumina Miseq PE300 sequencing platform (Illumina, Inc., CA, USA) according to the manufacturer’s recommendations.

### Sequence analysis

Raw sequencing data were processed by Beijing Auwigene Tech, Ltd. (Beijing, China) using the pipeline tools QIIME [11] and MOTHUR [12]. To retain only high-quality sequences for the downstream analysis, sequences that were less than 100 bp in length after quality trimming, contained one or more ambiguous base-calls (N), or had < 90% quality scores > Q20 were eliminated. After trimming, high-quality sequences were aligned to the Ribosomal Database Project [13] and were clustered into operational taxonomical units (OTU) using QIIME at a 97% similarity level. Before further analysis, singleton OTUs were removed.

Downstream analysis was performed in the microbiology laboratory of Peking University School of Stomatology. The Student’s *t*-test was used to compare alpha and beta diversities. Differences in the relative abundances of taxa within the PM, DM, DC, and PI samples were analyzed using the Kruskal—Wallis Test. P values have been corrected for pairwise comparisons. The Spearman correlation coefficients (SCC) were calculated for each pair of core genera. Edges were set between pairs of genera for which the SCC was >0.4 and significant (P < 0.05). We plotted differential features pairwise of the four groups and between permanent teeth (PT, including PM and PI samples) and deciduous teeth (DT, including DM and DC samples) using LEfSe [14]; the threshold logarithmic LDA score for discriminative features was 2.0.

## Results

### Overall sequence statistics

A total of 919 subjects were recruited in this study, of whom 142 were caries-free. Finally, 20 subjects met the inclusion criteria and underwent sample collection (Table 1 and S1 Table). Because sufficient dental plaque was not obtained from deciduous canines or/and incisors of four children, there were 16 samples for DC group. A total of 76 samples were collected for further analysis.

**Table 1:** Demographic characteristics of the subjects and sample size.

Demographic description of subjects	Sample size		20
	Ethnicity		Han
	Gender (male/ female)		11/9
	Age (months ± SD)		92.95 ± 10.08
	Plaque index (mean ± SD)		1.95±0.248
Sample size	PT	PM	20
	PT	PI	20
	DT	DM	20
	DT	DC	16

PT, permanent teeth; DT, deciduous teeth; PM, permanent molars; PI, permanent incisors; DM, deciduous molars; DC, deciduous canines or/and incisors.

The DNA amount recovered from clinical samples was 2.747 ± 1.23μg (range 0.424–6.535μg). In total, 1,371,906 high-quality sequences were generated after processing, with a mean of 18,051 ± 3,569 (range 7,012–26,100) per sample. We finally detected 19,914 OTUs, with 476–2,547 OTUs in individual specimens, using a 97% similarity level (for details see S1 Table).

### Microbial community composition

The diversity and composition of microbial communities within the mixed dentition were explored by comparing the PM, DM, DC, and PI sites.

**Fig 2:**
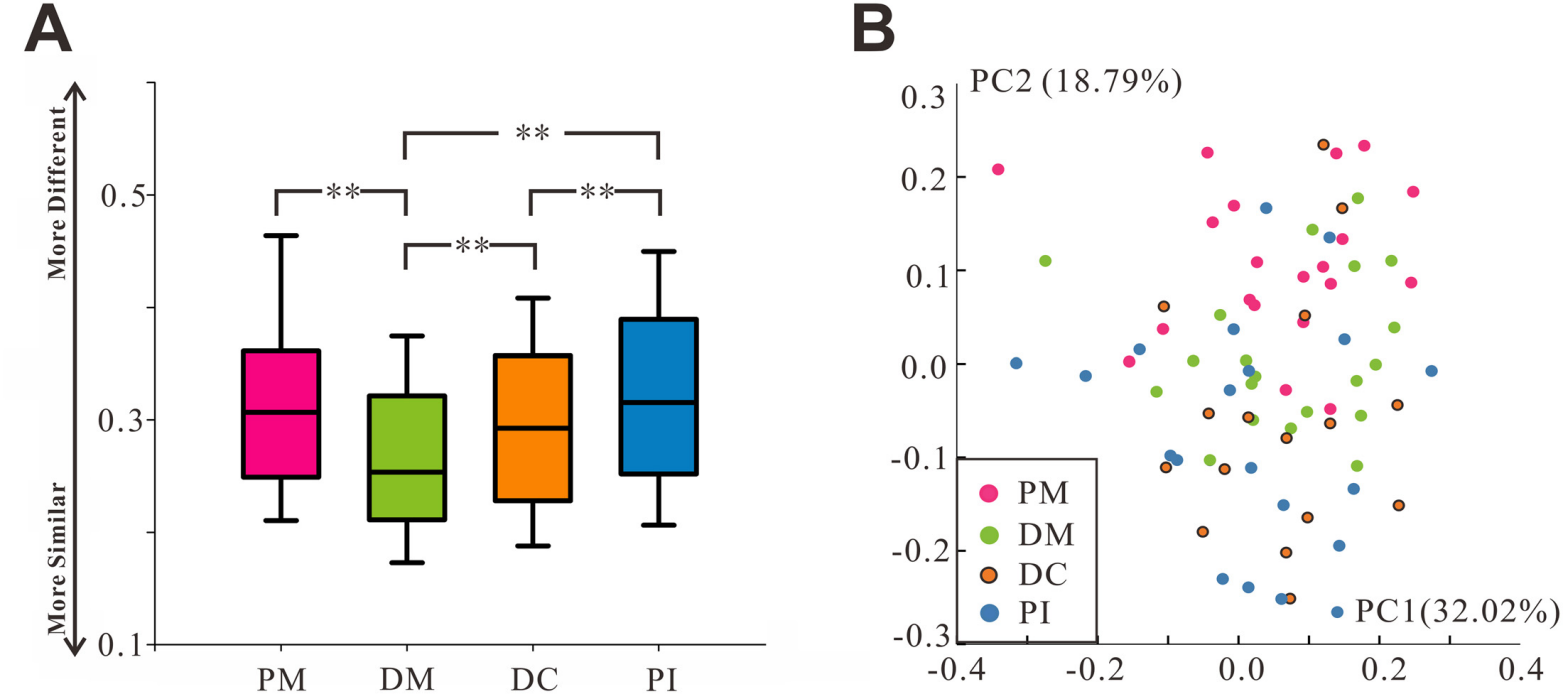
Microbial community variation within groups using weighted UniFrac distance. (A) The weighted UniFrac distance values of PM, DM, DC, and PI. PM and PI sites tended to host diverse bacterial communities, whereas DM and DC harbored similar microbial communities. All data are presented as medians and 10^th^, 25^th^, 75^th^, and 90^th^ percentiles. **P < 0.01 by two-tailed *t*-test. (B) A principal coordinate analysis (PCoA) based on the weighted UniFrac distance values.

The overall microbial community compositions based on weighted UniFrac distance measurements (beta diversities) were compared among the four groups (Fig 2). The microbial composition within each group differed less between the DM and DC sites and more between the PM and PI sites. There are statistically significant differences of microbial variation in comparison between any two groups with the exception of those between PM and DC groups and between PM and PI groups (Fig 2A). Additionally, the four groups showed relatively significant separation in the chart plotted based on principal coordinate analysis (PCoA) (Fig 2B). The microbial diversity within each sample (the alpha diversity) was calculated for given numbers of sequences (n = 10,000). Insufficient reads (<10,000 per sample) were obtained from one sample, which therefore was excluded from the alpha diversity analysis. The OTU richness (observed OTU index), evenness (equitability index), microbial diversity estimator (Shannon diversity index) and phylogenetic diversity were explored separately. Most alpha diversity values showed no significant differences among the groups (S1 Fig).

The microbial community composition was characterized by the relative abundances of microbial taxa. A total of 12 phyla, 21 classes, 38 orders, 66 families, and 74 genera were detected in the supragingival dental plaque samples.

**Fig 3:**
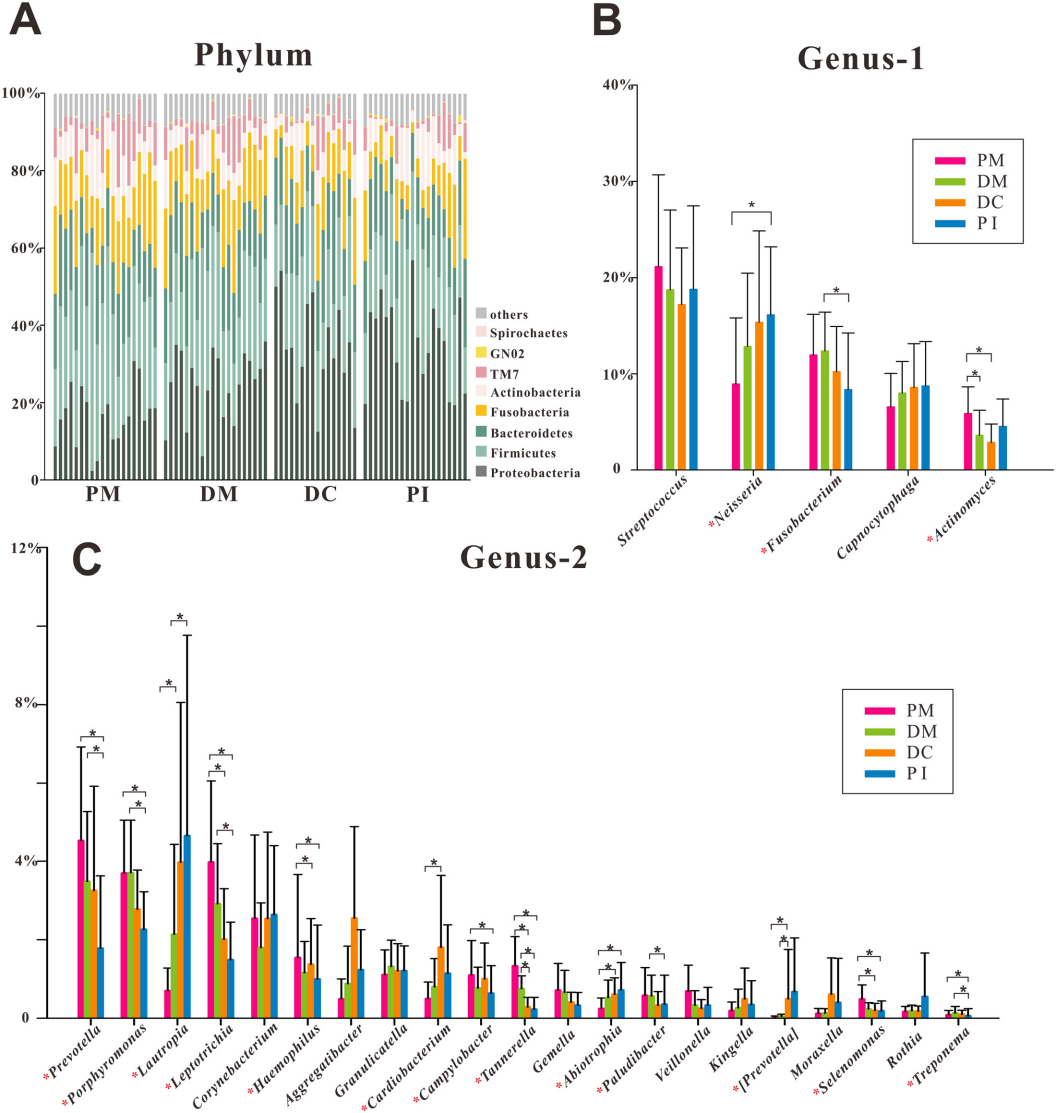
Bacterial composition of supragingival plaque samples. (A). Relative abundances of the resident bacterial phyla at various sampling sites. (B, C). Relative bacterial abundance at the genus level. Genera with a mean relative abundance value >0.1% are shown. Bars indicate mean relative abundances with standard deviations. The significance of differences among groups was determined using the Kruskal—Wallis test. *P < 0.05.

**Fig 4:**
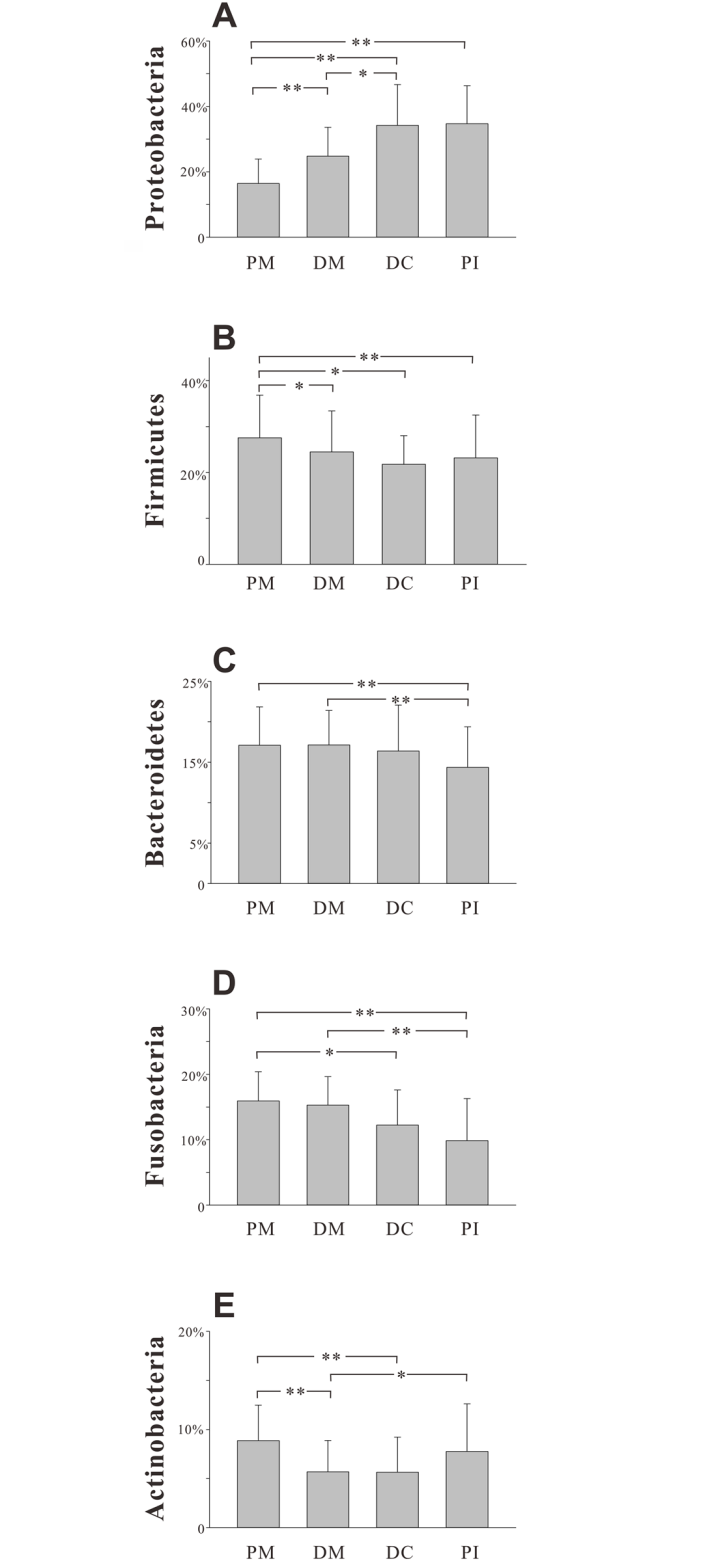
Relative abundances of the main bacterial phyla identified in dentition sites. A–E. Relative abundances of Proteobacteria, Firmicutes, Bacteroidetes, Fusobacteria and Actinobacteria in the four groups. The four groups are presented in distal—mesial order according to location in the dentition. All data are presented as means with standard deviations. *P < 0.05, **P < 0.01 by two-tailed *t*-test.

At the phylum level, the vast majority of the sequences (>90%) belonged to one of the following five phyla (Fig 3A): Proteobacteria (2–56% of total sequences), Firmicutes (11–56%), Bacteroidetes (5–29%), Fusobacteria (3–26%) and Actinobacteria (2–17%). Although these five phyla were detected in all samples, the relative abundance of each was highly variable among sites (Fig 4). The remaining microbiota belonged to Spirochaetes, Tenericutes, Synergistetes, Nitrospirae, candidate division TM7, GN02 or SR1.

A total of 74 genera were detected. We selected and compared those genera with mean relative abundance values >0.1%; 16/26 genera showed significant differences in relative abundance among the groups (Fig 3B and 3C).

The between-group microbial variation was explored at the phylum, class, order, family, and genus levels separately using the LEfSe online tool. Differences in microbial community composition were observed between pairwise groups (S2 Fig).

### Permanent and deciduous teeth harbor distinct but related microbial communities

**Fig 5:**
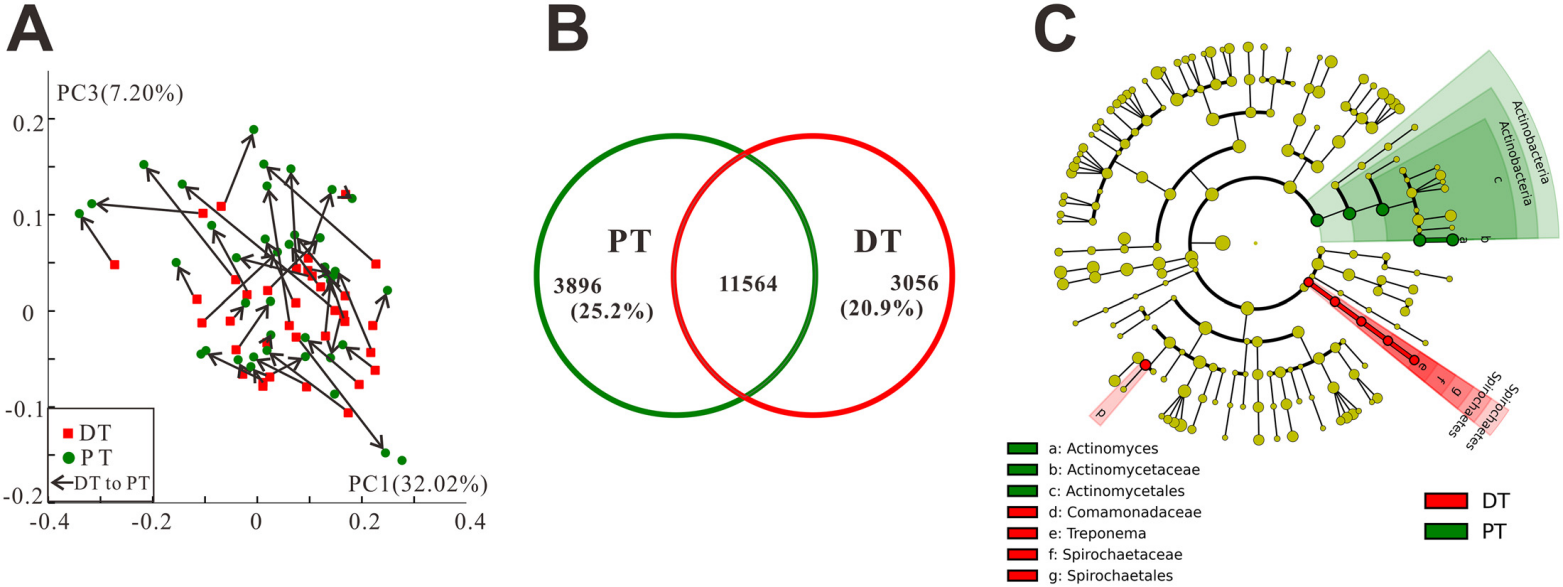
Microbial communities in permanent teeth (PT) and deciduous teeth (DT). (A) A principal coordinate analysis (PCoA) based on weighted UniFrac distances suggested similar relationships among the 76 samples. Samples collected from the same individuals are shown as connected by arrows. The DT→PT pairs consisted of DM→PM and DC→PI pairs since DM and PM, DC and PI had similar morphological characteristics (four free PI dots resulted from four missing DC samples). Most marked arrows displayed a trend in the negative to positive direction of PC3, suggesting the possibility of an analogical microbial transformation upon replacement of teeth in the mixed dentition. (B) Venn diagram of the shared and unique OTUs between PT and DT. Of the total OTUs, 25.2% and 20.9% were unique to PT and DT, respectively. (C) The microbial composition variation was compared using the LEfSe online tool. The difference in the microbial community at the genus level was due mainly to *Actinomyces* and *Treponema*.

A principal coordinate analysis (PCoA) based on weighted UniFrac distance demonstrated similar relationships between the permanent and deciduous teeth samples. Samples collected from the same individuals are shown as connected by arrows. Most marked arrows displayed a trend in the negative to positive direction of principal coordinate (PC) 3 (Fig 5A), suggesting the possibility of an analogical process in microbial transformation among children during tooth replacement in the mixed-dentition stage.

The difference in microbial composition between permanent and deciduous teeth was reflected by the shared OTUs of samples collected from these two sites. More than 20% of the total OTUs were detected only in permanent teeth (25.2%) or deciduous teeth (20.9%) (Fig 5B). The variation in microbial community composition between the two groups at the phylum, class, order, family and genus levels was assessed using the LEfSe online tool (Fig 5C). The significant differences in microbial community composition were due mainly to the enrichment of permanent teeth in *Actinomyces* and deciduous teeth in *Treponema*.

### The core microbiome of supragingival plaque

A fundamental question regarding the oral microbiome of healthy individuals is the presence of an identifiable common ‘core microbiome’ [15]. We tested this hypothesis using a definition described previously [3] at the phylum, class, order, family and genus levels (Table 2).

**Table 2:** Taxa common among the PM, DM, DC and PI sites in mixed dentition.

	Phylum	Class	Order	Family	Genus	OTU
Shared taxa (No. / %)	6/50%	10/47.6%	13/34.2%	23/34.8%	19/25.7%	51/0.2%
Total relative abundance	90.26% -98.79%	90.26% -98.77%	88.57% -98.64%	87.37% -98.3%	86.03% -96.97	47.71% -84.04%

No., number of shared taxa; %, percentage of total taxa.

At the phylum level, 50% (6/12) of the total phyla could be detected in all 76 samples and accounted for 90.26–98.79% of the entire microbial community. Although only 25.7% of total genera and 0.2% of total OTUs were shared among samples, they accounted for 86.03–96.97% and 47.71–84.04% of sequences obtained at the genus and OTU levels, respectively.

**Fig 6:**
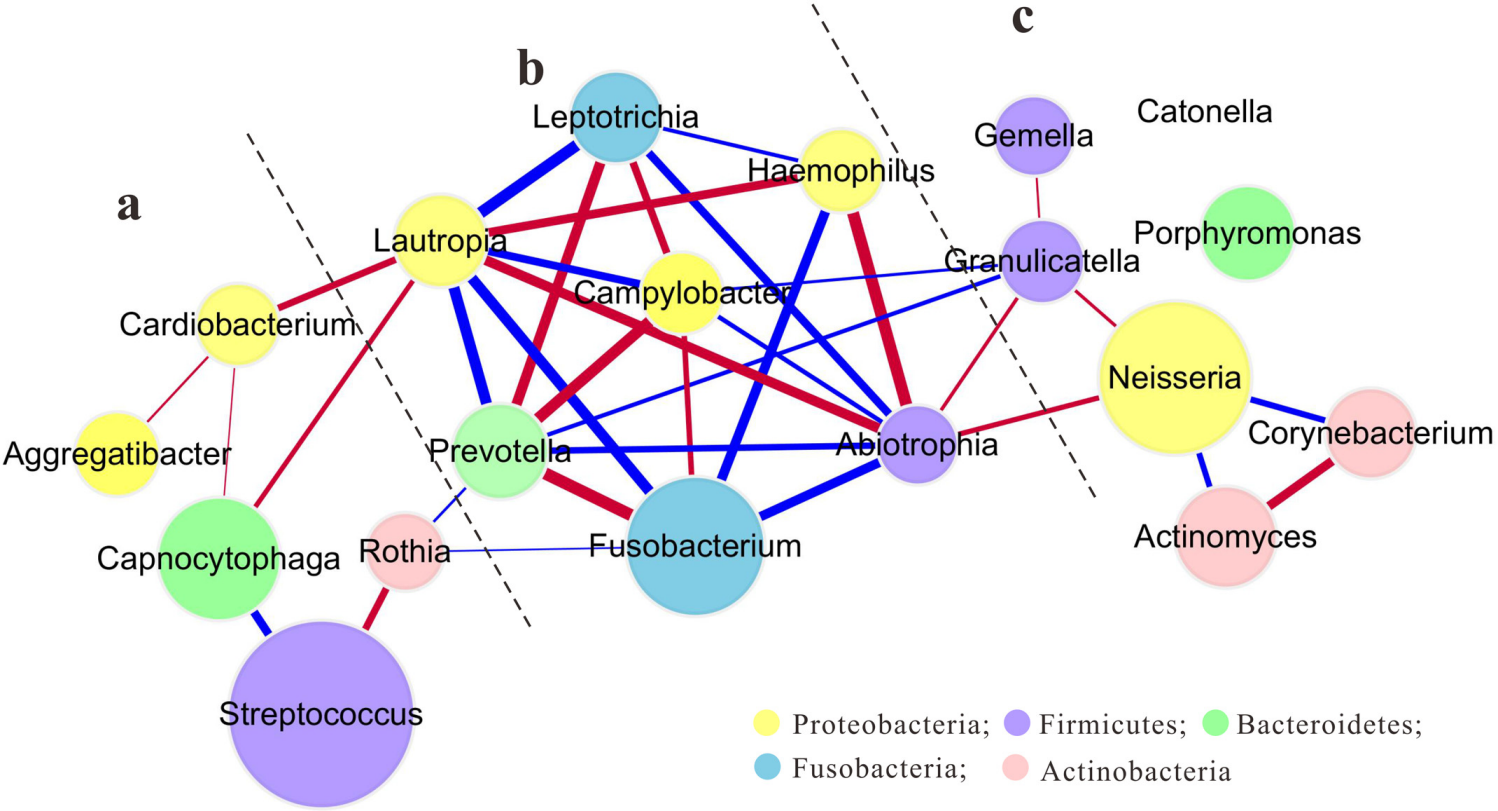
“Core microbiome” in the mixed dentition. The dots represent genera that belong to the “core microbiome”. The dot size indicates the mean relative abundance of the corresponding genus in all samples. Colors indicate the different phyla. Lines link two genera having coefficient values >0.4. Line thickness indicates the coefficient value. Red and blue lines indicate positive and negative correlationships, respectively. From a macroscopic perspective, this core microbiome could be divided into a relatively simple (a, c) and a relatively complex interaction component (containing the *Lautropia—Haemophilus—Abiotrophia* and *Prevotella—Fusobacterium—Leptotrichia—Campylobacter* groups, b).

The core microbiome at the genus level contained 19 genera, which were rendered into a co-occurring network module according to their Spearman correlation coefficient (SCC) values (Fig 6). The core microbial community showed both positive and negative SCC values, the degrees of which differed. High SCC values were found for the *Prevotella—Fusobacterium*, *Abiotrophia—Haemophilus*, and *Leptotrichia—Lautropia* pairs, while *Porphyromonas* and *Catonella* exhibited poor correlationships with other genera.

## Discussion

Our findings demonstrated considerable differences in microbial diversity and composition between the permanent and deciduous teeth in mixed dentition.

A possible reason for this is the gradients and variations in physicochemical factors, *e*.*g*., oxygen, at different locations within the oral cavity [16]. This was due to the distal-mesial downtrend in the average relative abundance of obligate or facultative anaerobes, such as Fusobacteria and Bacteroidetes (Fig 4C and 4D,S4C and S4D Fig). However, environmental factors could not explain all of our findings. For example, the first permanent molars are adjacent to the deciduous molars in this mixed dentition. Thus, despite existing in a similar physicochemical environment, the two molar types exhibited different microbial compositions in this study.

The residence time in the oral cavity may also affect the microbial composition. In this study, the microbial communities of sites in deciduous teeth (DM and DC) were more similar than those in permanent teeth (PM and PI) (Fig 2A). The deciduous teeth had been in place for ~4–6 years, compared with ~0.5–2 years for the permanent teeth. A potential explanation is that compared with the newly erupted permanent teeth, the deciduous teeth have had greater opportunity for development and maturation. Previous studies have reported temporal shifts in the microbial communities of single teeth [6] and pooled plaque [17].

Another major factor contributing to the microbial variation within the mixed dentition may be the different surface structure of permanent and deciduous teeth. The surface structure, such as microstructure and mineral composition of dental enamel, was different between permanent teeth and deciduous teeth [18, 19]. For example, De Menezes Oliveira et al [20] demonstrated that numerical density of enamel rods was higher in the deciduous teeth and the percentage of Ca and P was higher in the permanent teeth enamel. In our study, *Actinomyces* abundance differed significantly between permanent and deciduous teeth. *Actinomyces* is an initial colonizer during dental plaque biofilm formation [21, 22] and may attach directly to the acquired enamel pellicle [23]. Zimmerman *et al*. [24] reported that the proteome and peptidome of acquired enamel pellicles differed between deciduous and permanent teeth. Therefore, it is possible that fluctuations in acquired enamel pellicles composition mediate the effect of surface structure on the microbial communities of teeth.

In addition to the differences in microbial communities between permanent and deciduous teeth, we explored the shared microbiota, the “core microbiome”, within the mixed dentition. Identification of a core microbiome is crucial for understanding the stable, consistent components of complex microbial assemblages. Xu *et al*. [3] proposed that the core oral microbiome should be defined according to age and location within the oral cavity. Accordingly, an aim of our study was to describe the supragingival core microbiome among sites of permanent and deciduous teeth in mixed dentition. The core microbiome was comprised of 19 genera (Fig 6) detected in all samples. With the exceptions of *Lautropia*, *Aggregatibacter*, *Haemophilus*, *Rothia*, and *Catonella*, the remaining 14 genera were consistent with those reported previously in the core community at this dentition stage [3]. We then explored and compared the “sub-core” microbiome of the permanent and deciduous teeth in the mixed dentition-stage children. Besides 19 genera in “core microbiome”, the “sub-core” microbiome of the permanent teeth also contained *Veillonella*, *Selenomonas*, *Paludibacter* and that of the deciduous teeth included an extra *Tannerella*.

The correlationships among these 19 genera were then explored. The resultant complex correlationship network may be caused by the diversity of bacterial interactions taking place within the oral microbiome. These interactions occur at several levels, including physical contact, metabolic exchange, small signaling molecule-mediated communication and exchange of genetic material [22, 25], and are important for the formation of dental biofilm. For example, the *Prevotella—Fusobacterium* pair exhibited the highest positive correlation coefficient value in this study. This phenomenon is consistent with their coaggregation reaction [26], which is thought to be important during dental biofilm formation [27] and is compatible with their similar aciduric ability, glucose utilization and acid-neutralizing activity [28]. The *Abiotrophia—Haemophilus* pair had the second highest correlation coefficient value. Few studies were related to the interactions of *Abiotrophia* and *Haemophilus*. A possible mechanism for the correlation is that both *Abiotrophia* and *Haemophilus*, nutritionally deficient genera, are unable to grow on blood agar, because they lack a required factor. In the case of *Haemophilus*, the missing factor is nicotinamide adenine dinucleotide (NAD, factor V) or heme (factor X) [29], and for *Abiotrophia*, a source of pyridoxal must be provided [30]. Some bacteria, such as *Staphylococcus aureus*, secrete both NAD and pyridoxal. This permits the growth of both *Abiotrophia* and *Haemophilus* on blood agar as satellite colonies [31]. More studies should be required to test this assumption and explore other possible mechanisms of this correlationship. From a macroscopic perspective, this core microbiota could be divided into a relatively simple (Fig 6a–6c) and a relatively complex correlation component (containing the *Lautropia—Haemophilus—Abiotrophia* and *Prevotella—Fusobacterium—Leptotrichia—Campylobacter* groups, Fig 6b). The interpretation of this phenomenon should be the focus of further research.

According to the third national oral health survey, the caries prevalence rate is 64.5% among 5-year-olds in Hunan Province, China where our study subjects reside [32]. But in our study, only 15.45% of the children investigated did not have clinical manifestation of dental caries. One of the reasons may be that our study subjects were generally around 7–9 years old. Comparing with 5-year-olds in the national oral health survey, they inclined to have more opportunities with dental caries, especially with caries of primary molars and the first permanent molars. Furthermore, dental caries in our study were charted using the International Caries Detection and Assessment System (ICDAS) instead of WHO criteria which were used in the third national oral health survey. This could be another reason of higher caries prevalence in our study. Some of these caries-free children had more/less than 10–12 permanent teeth, visually or clinically detected gingivitis, insufficient supragingival plaque samples, non-cooperation activities, recent antibiotic use or fluoride treatment and so on. Finally, only 20 subjects met the inclusion criteria and underwent sample collection.

The present results indicate that microbial diversity and community composition differ among permanent and deciduous tooth sites in the mixed dentition. Additionally, the core microbiome of these sites was determined. These findings enhance our understanding of the development of the native oral microbiota with age.

The English in this document has been checked by at least two professional editors, both native speakers of English. For a certificate, please see: http://www.textcheck.com/certificate/Qfx7Fu

## Supporting Information

S1 Fig Calculation of alpha diversity values for comparison of the total microbial diversity of the first permanent molars (PM), deciduous molars (DM), deciduous canines and incisors (DC) and permanent incisors (PI) sites. (A) The observed OTU index is used to describe OTU richness. Compared with plaque samples from DC, samples from DM showed higher OTU richness. (B, C) Equitability and the Shannon index are conventional values of community evenness and diversity, respectively. No significant difference was evident among the groups. (D) Phylogenetic diversity (PD) is a measure of community diversity; the microbial communities of DM sites were more diverse than those of DC and PI sites.
(PDF)

S2 Fig Pairwise comparison of microbial communities among the first permanent molars group (PM), deciduous molars group (DM), deciduous canines and incisors group (DC) and the permanent incisors group (PI) using the LEfSe online tool. (PDF)

S3 Fig Agarose gel electrophoresis of microbial genomic DNA. (PDF)

S4 Fig Mean relative abundances of the main bacterial phyla identified in dentition sites. (PDF)

S1 Table Basic information of subjects and sequencing results. (DOC)
