# Supplementary figures and images for: Supragingival Microbial Profiles of Permanent and Deciduous Teeth in Children with Mixed Dentition
Source: PLoS One. 2016 Jan 11;11(1):e0146938. doi: 10.1371/journal.pone.0146938 (PMC4709228; doi:10.1371/journal.pone.0146938)

**A**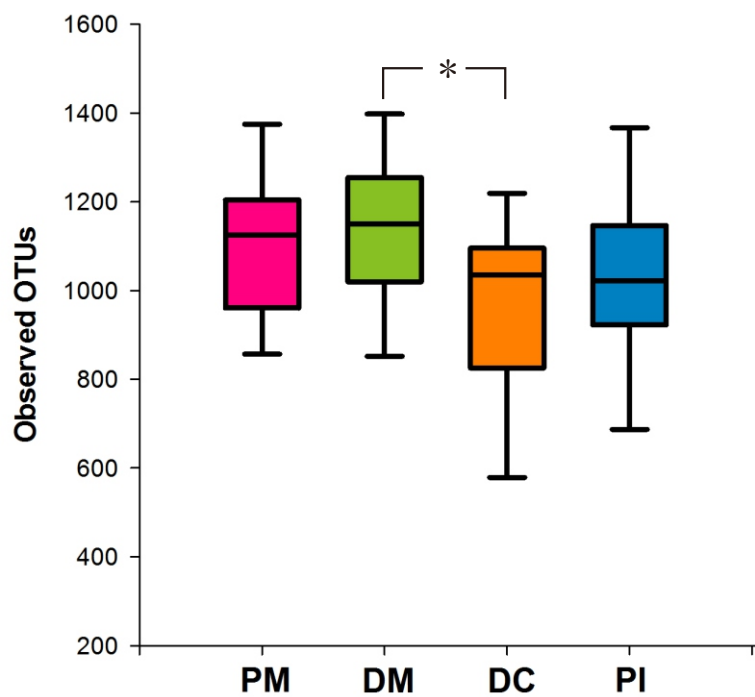**B**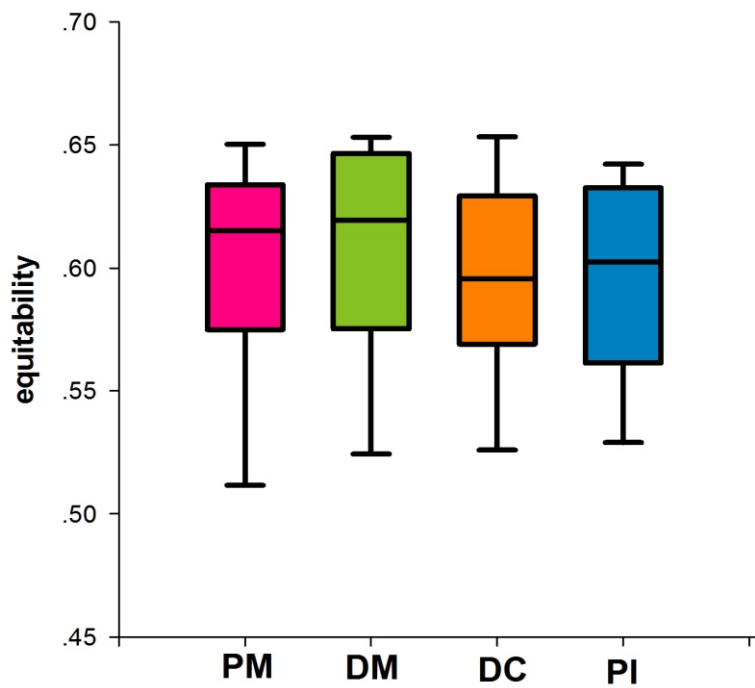**C**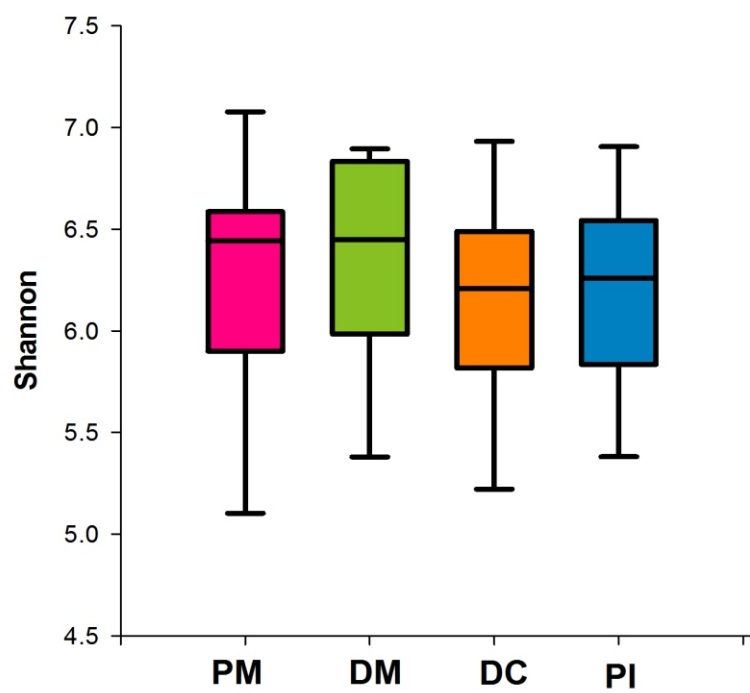**D**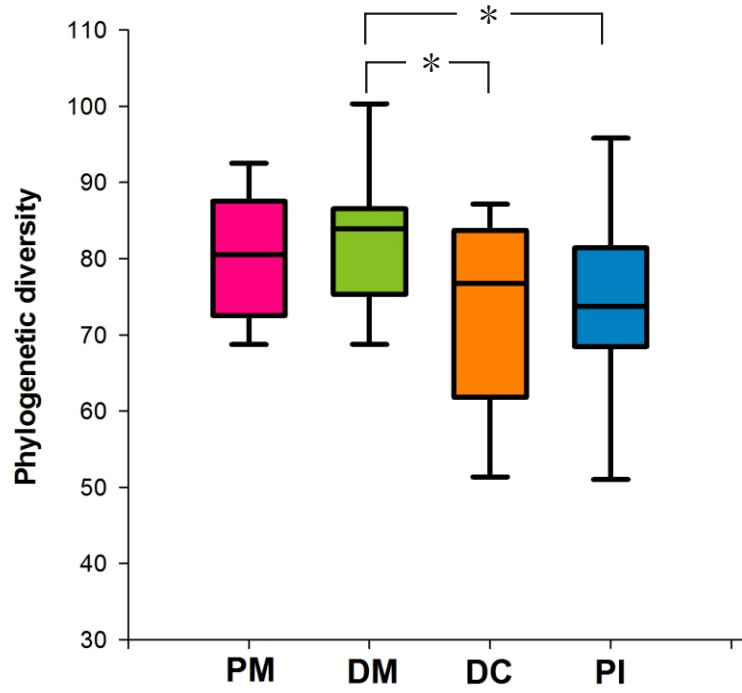

Supplement: S1 Fig — (A) The observed OTU index is used to describe OTU richness. Compared with plaque samples from DC, samples from DM showed higher OTU richness. (B, C) Equitability and the Shannon index are conventional values of community evenness and diversity, respectively. No significant difference was evident among the groups. (D) Phylogenetic diversity (PD) is a measure of community diversity; the microbial communities of DM sites were more diverse than those of DC and PI sites. (PDF) [file pone.0146938.s001.pdf]

# A

DM  
PM

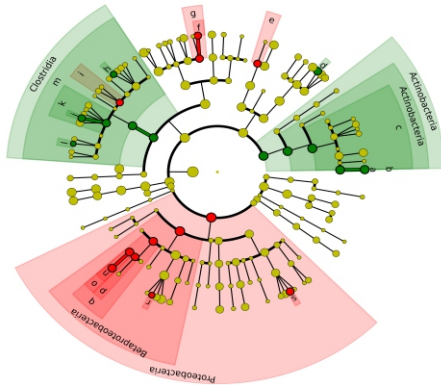

# B

DC  
PM

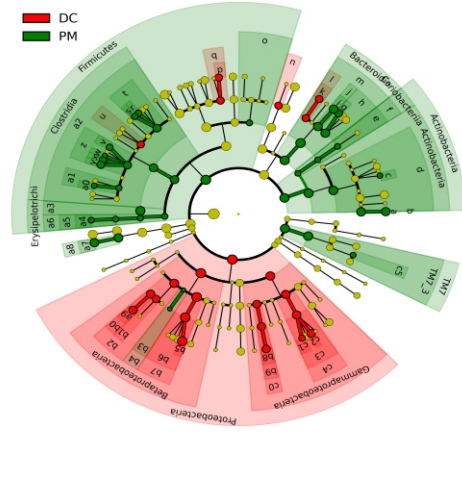

# C

PI  
PM

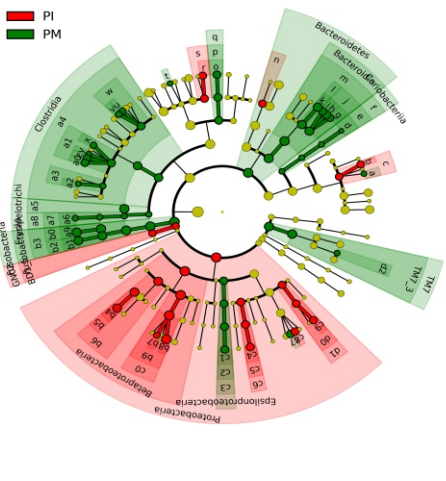

# D

DM  
PI

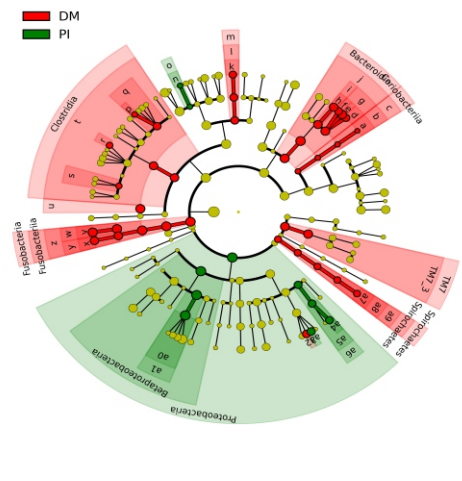

# E

DC  
DM

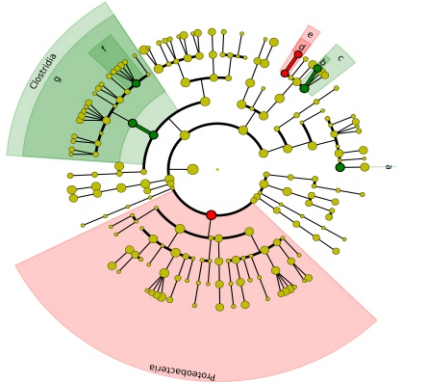

# F

DC  
PI

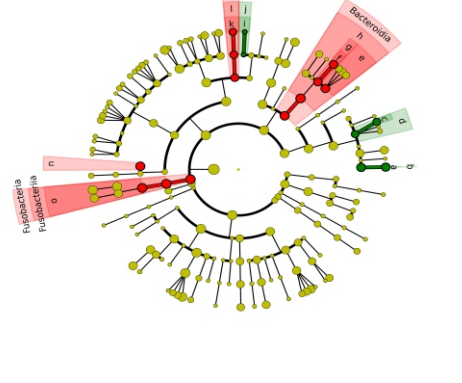

Supplement: S2 Fig — (PDF) [file pone.0146938.s002.pdf]

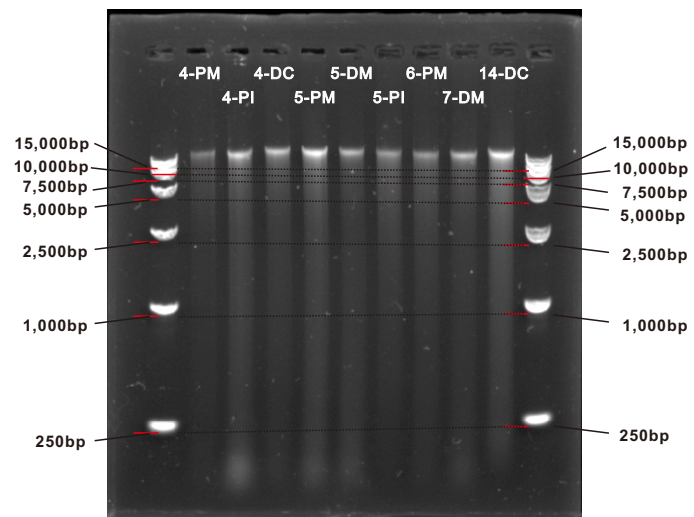

Supplement: S3 Fig — (PDF) [file pone.0146938.s003.pdf]

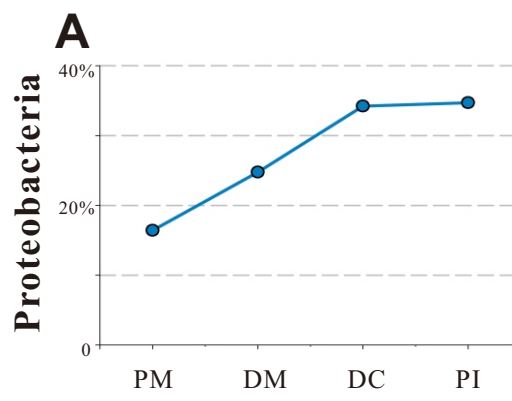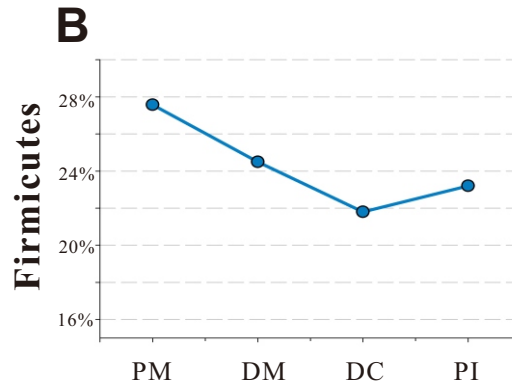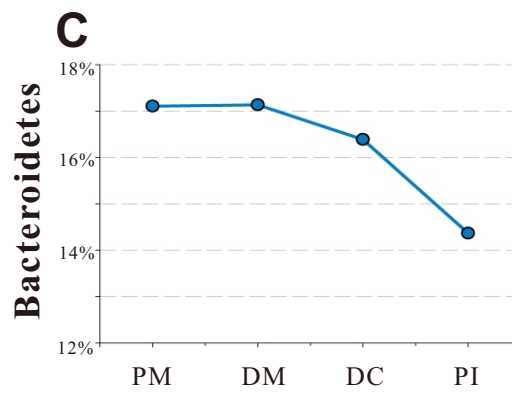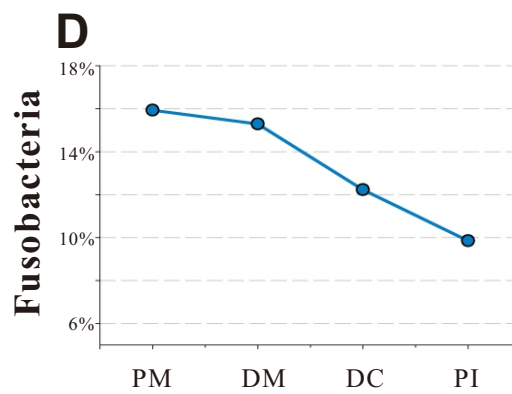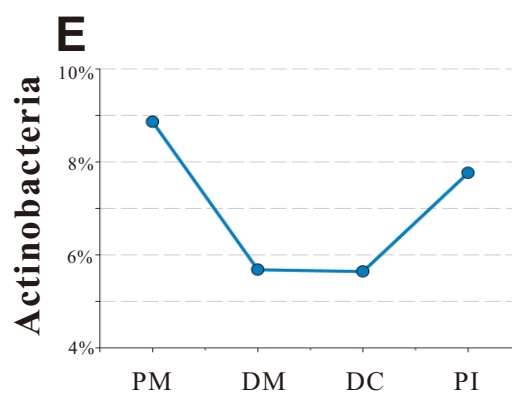

Supplement: S4 Fig — (PDF) [file pone.0146938.s004.pdf]
