# Supplementary material for: Supragingival Microbial Profiles of Permanent and Deciduous Teeth in Children with Mixed Dentition
Source: PLoS One. 2016 Jan 11;11(1):e0146938. doi: 10.1371/journal.pone.0146938 (PMC4709228; doi:10.1371/journal.pone.0146938)
Supplement: S1 Table — (DOC) [file pone.0146938.s005.doc]

**S1 Table: Basic information of subjects and sequencing results**

| **Sample** | **Gender**  **(M/F)** | **Age**  **(months)** | **Ethnicity** | **Nr of**  **PT** | **Nr of**  **DT** | **Group** | **Genomic DNA amount (μg)** ***** | **Sequences** | **OTUs** | **Plaque index**** |
| --- | --- | --- | --- | --- | --- | --- | --- | --- | --- | --- |
| **1** | F | 79 | Han | 10 | 14 | PM | 4.3145 | 13884 | 1805 | 2.17 |
|  |  |  |  |  |  | DM | 4.581 | 13180 | 1745 |  |
|  |  |  |  |  |  | DC | - | - | - |  |
|  |  |  |  |  |  | PI | 2.3635 | 11980 | 1689 |  |
| **2** | F | 89 | Han | 12 | 12 | PM | 2.4655 | 14843 | 1818 | 2.00 |
|  |  |  |  |  |  | DM | 2.1245 | 16624 | 1826 |  |
|  |  |  |  |  |  | DC | 1.221 | 14277 | 1298 |  |
|  |  |  |  |  |  | PI | 2.5845 | 14302 | 1400 |  |
| **3** | F | 94 | Han | 10 | 14 | PM | 1.727 | 17170 | 1767 | 1.83 |
|  |  |  |  |  |  | DM | 2.4805 | 17789 | 1702 |  |
|  |  |  |  |  |  | DC | 2.283 | 20567 | 1269 |  |
|  |  |  |  |  |  | PI | 2.3685 | 13745 | 1010 |  |
| **4** | F | 92 | Han | 10 | 14 | PM | 1.431 | 21422 | 1727 | 2.00 |
|  |  |  |  |  |  | DM | 1.8895 | 25056 | 2241 |  |
|  |  |  |  |  |  | DC | 1.5505 | 18847 | 1642 |  |
|  |  |  |  |  |  | PI | 4.82 | 15902 | 1306 |  |
| **5** | M | 83 | Han | 10 | 14 | PM | 3.479 | 18720 | 1803 | 2.17 |
|  |  |  |  |  |  | DM | 2.8505 | 22894 | 2547 |  |
|  |  |  |  |  |  | DC | 0.4436 | 23302 | 2235 |  |
|  |  |  |  |  |  | PI | 1.2875 | 19851 | 1924 |  |
| **6** | M | 78 | Han | 12 | 12 | PM | 1.4855 | 14455 | 1601 | 1.50 |
|  |  |  |  |  |  | DM | 1.585 | 19344 | 1939 |  |
|  |  |  |  |  |  | DC | - | - | - |  |
|  |  |  |  |  |  | PI | 1.706 | 15416 | 1509 |  |
| **7** | M | 86 | Han | 10 | 14 | PM | 1.6535 | 19627 | 2288 | 2.00 |
|  |  |  |  |  |  | DM | 2.2255 | 19649 | 2140 |  |
|  |  |  |  |  |  | DC | - | - | - |  |
|  |  |  |  |  |  | PI | 3.5795 | 21834 | 2420 |  |
| **8** | M | 104 | Han | 10 | 14 | PM | 3.38 | 15630 | 1242 | 1.67 |
|  |  |  |  |  |  | DM | 3.091 | 18107 | 1553 |  |
|  |  |  |  |  |  | DC | 0.4235 | 15683 | 1413 |  |
|  |  |  |  |  |  | PI | 3.6655 | 16825 | 1409 |  |
| **9** | M | 105 | Han | 12 | 12 | PM | 3.385 | 13152 | 1465 | 2.00 |
|  |  |  |  |  |  | DM | 3.418 | 15297 | 1577 |  |
|  |  |  |  |  |  | DC | 1.083 | 18116 | 1552 |  |
|  |  |  |  |  |  | PI | 3.7235 | 15637 | 1469 |  |
| **10** | M | 97 | Han | 10 | 14 | PM | 4.7125 | 16153 | 1407 | 1.67 |
|  |  |  |  |  |  | DM | 3.2335 | 7012 | 508 |  |
|  |  |  |  |  |  | DC | 2.9915 | 18057 | 999 |  |
|  |  |  |  |  |  | PI | 1.7215 | 18860 | 1083 |  |
| **11** | F | 98 | Han | 10 | 14 | PM | 1.743 | 17462 | 1280 | 1.67 |
|  |  |  |  |  |  | DM | 4.253 | 17350 | 1697 |  |
|  |  |  |  |  |  | DC | 1.1745 | 20201 | 1775 |  |
|  |  |  |  |  |  | PI | 2.817 | 19170 | 1936 |  |
| **12** | F | 93 | Han | 10 | 14 | PM | 1.475 | 23701 | 2194 | 2.17 |
|  |  |  |  |  |  | DM | 2.619 | 20785 | 2345 |  |
|  |  |  |  |  |  | DC | 0.7015 | 26100 | 2065 |  |
|  |  |  |  |  |  | PI | 1.0495 | 22077 | 1835 |  |
| **13** | F | 86 | Han | 12 | 12 | PM | 3.0265 | 25269 | 1923 | 2.17 |
|  |  |  |  |  |  | DM | 2.425 | 18782 | 1875 |  |
|  |  |  |  |  |  | DC | 1.0025 | 19497 | 1927 |  |
|  |  |  |  |  |  | PI | 2.5485 | 15652 | 1421 |  |
| **14** | M | 96 | Han | 10 | 14 | PM | 3.2695 | 20559 | 1782 | 1.67 |
|  |  |  |  |  |  | DM | 3.443 | 21110 | 1821 |  |
|  |  |  |  |  |  | DC | 1.653 | 16271 | 959 |  |
|  |  |  |  |  |  | PI | 3.696 | 15642 | 1267 |  |
| **15** | M | 94 | Han | 10 | 14 | PM | 4.452 | 13216 | 1085 | 2.17 |
|  |  |  |  |  |  | DM | 4.9055 | 17768 | 1626 |  |
|  |  |  |  |  |  | DC | 3.759 | 17645 | 1516 |  |
|  |  |  |  |  |  | PI | 3.612 | 20687 | 1492 |  |
| **16** | M | 90 | Han | 12 | 12 | PM | 2.6865 | 17317 | 1449 | 2.50 |
|  |  |  |  |  |  | DM | 2.3925 | 20967 | 1566 |  |
|  |  |  |  |  |  | DC | 2.6605 | 12904 | 476 |  |
|  |  |  |  |  |  | PI | 5.72 | 17282 | 726 |  |
| **17** | M | 95 | Han | 10 | 14 | PM | 3.7835 | 11463 | 551 | 2.00 |
|  |  |  |  |  |  | DM | 4.558 | 10878 | 515 |  |
|  |  |  |  |  |  | DC | 3.088 | 20228 | 1768 |  |
|  |  |  |  |  |  | PI | 6.535 | 22046 | 1686 |  |
| **18** | M | 92 | Han | 11 | 12 | PM | 4.2 | 20084 | 1751 | 2.00 |
|  |  |  |  |  |  | DM | 3.3395 | 15500 | 1194 |  |
|  |  |  |  |  |  | DC | 1.3035 | 22949 | 2051 |  |
|  |  |  |  |  |  | PI | 3.236 | 22459 | 1871 |  |
| **19** | F | 123 | Han | 12 | 12 | PM | 2.7085 | 19901 | 2002 | 2.00 |
|  |  |  |  |  |  | DM | 3.3075 | 19782 | 1567 |  |
|  |  |  |  |  |  | DC | 3.1165 | 18811 | 1510 |  |
|  |  |  |  |  |  | PI | 2.5155 | 18602 | 1527 |  |
| **20** | F | 85 | Han | 10 | 14 | PM | 2.0325 | 21896 | 1992 | 1.67 |
|  |  |  |  |  |  | DM | 2.839 | 19442 | 1946 |  |
|  |  |  |  |  |  | DC | - | - | - |  |
|  |  |  |  |  |  | PI | 1.826 | 17242 | 1743 |  |

PT, permanent teeth; DT, deciduous teeth;

* Genomic DNA amount: calculate according to the concentration showed by an ultraviolet spectrophotometer and the final solution volumes

**Plaque index：Average score of surfaces scored.
